# Mitochondrial DNA drives neuroinflammation through the cGAS-IFN signaling pathway in the spinal cord of neuropathic pain mice

**DOI:** 10.1515/biol-2022-0872

**Published:** 2024-05-31

**Authors:** Penghui Huang, Li Li, Yaohua Chen, Yuping Li, Dan Zhu, Jian Cui

**Affiliations:** Department of Pain Medicine, First Affiliated Hospital, Army Medical University, Chongqing, 400038 China

**Keywords:** neuropathic pain, microglia, mitochondrial deoxyribonucleic acid, neuroinflammation

## Abstract

Neuroinflammation is pivotal in the development of neuropathic pain (NeP). While mitochondrial deoxyribonucleic acid (mtDNA) and cyclic GMP-AMP synthase (cGAS) are recognized for inducing inflammation in various neurological disorders, their involvement in NeP remains ambiguous. In this study, we examined: (1) the changes in mtDNA and cGAS in mice with NeP induced by chronic constriction injury (CCI) of the sciatic nerve, whether mtDNA triggers inflammation via the cGAS signaling; (2) the effects of RU.521, a cGAS antagonist, on CCI-induced nociception (allodynia and hyperalgesia) and relative inflammatory protein expression; (3) the activation of microglia and the cGAS-IFN pathway mediated by mtDNA in BV2 cell; (4) the effect of RU.521 on mtDNA-induced inflammatory response in BV2 cells. Results revealed reduced mtDNA levels in the sciatic nerve but increased levels in the spinal cord of CCI mice, along with elevated cGAS expression and inflammatory factors. RU.521 alleviated nociceptive behaviors in CCI mice, possibly by normalizing cGAS levels and suppressing inflammation. Neuron-derived mtDNA provoked cellular activation and upregulated cGAS signaling in BV2 cells. Additionally, RU.521 and DNase I effectively inhibited cGAS-induced inflammation. These findings underscore the critical role of mtDNA accumulation and mtDNA-mediated cGAS signaling in NeP development after peripheral nerve injury.

## Graphical abstract

**Figure:**
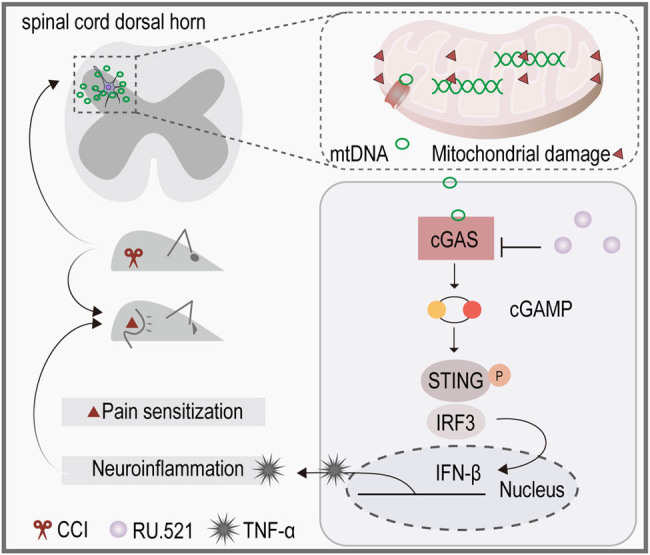

## 1 Introduction

Neuropathic pain (NeP) presents as a prevalent and formidable clinical challenge, characterized by clinical manifestations such as allodynia and hyperalgesia, impacting approximately 10% of the world population [1,2]. Nevertheless, its underlying causes remain poorly understood. Our earlier research has substantiated that mitochondrial dysfunction contributes to the advancement of NeP by promoting neuroinflammation and oxidative stress [3]. Additionally, enhancing mitochondrial autophagy can alleviate hyperalgesia by reducing neuroinflammation [4,5]. However, the specific mechanism by which mitochondrial dysfunction triggers neuroinflammation in the NeP remains to be further elucidated.

Recently, increasing evidence suggests that mitochondrial deoxyribonucleic acid (mtDNA) plays a significant role in the pathogenesis of various neurological diseases [6,7], including amyotrophic lateral sclerosis [8], multiple sclerosis (MS) [9], and ischemic stroke [10]. Besides, previous studies have demonstrated that elevated reactive oxygen species (ROS) not only impair mitochondrial function but also induce oxidative stress reactions that damage various biomolecules such as DNA, RNA, lipids, and proteins [6,11]. The oxidative stress leads to the release of mtDNA into the cytoplasm and intercellular space, thereby initiating immune responses [12]. However, the impact of mtDNA on NeP and the possible mechanisms involved warrant further investigation.

The mtDNA fragments can directly bind to pattern recognition receptors on the cellular membrane, initiating the signaling cascade, and activating immune-related genes [12,13]. In addition, the release of mtDNA also activates the Cyclic GMP-AMP synthase (cGAS) pathway [14,15]. The cGAS is a DNA sensor present in the cytoplasm and on the plasma membrane [16], it is capable of binding to mtDNA as a damage-associated molecular pattern due to its distinctive molecular structure. Then their combination catalyzes ATP and GTP to generate the secondary messenger 2′–3′ cyclic GMP-AMP (2′–3′ cGAMP), facilitating the translocation and phosphorylation of the adaptor protein known as stimulator of interferon genes (STING). Subsequently, the activated STING triggers the activation of TGFβ-activated kinase 1 and the transcription factor interferon regulatory factor 3 (IRF3), which play important roles in the innate immune system’s response [17,18]. This chain of events ultimately leads to the enhancement of type I interferon (IFN) response, resulting in inflammation in response to viral and microbial infections [19,20]. Even though the activated cGAS exhibits obvious immunomodulatory effects, it is still unknown whether mtDNA triggers inflammatory responses through the cGAS signaling pathway in NeP.

In this study, we assessed alterations in mtDNA and cGAS levels and the relevant inflammatory responses in the spinal cord and/or sciatic nerve of NeP mice induced by chronic constriction injury of the sciatic nerve (CCI) and explored the antinociceptive effect of the administration of cGAS inhibitor. Subsequently, we conducted *in vitro* experiments to appraise the impact of neuron-derived mtDNA on microglial activation, and whether the inhibition of cGAS could mitigate the inflammatory response.

## 2 Materials and methods

### 2.1 Animals

C57BL6/J male mice (8–12 weeks of age) were purchased from Hunan SJA Laboratory Animal Co., Ltd (Hunan, China). All experimental procedures were approved by the Laboratory Animal Welfare and Ethics Committee of the Army Medical University and followed the principle of minimizing animal suffering, as few as possible the animals used.


**Ethical approval:** The research related to animal use has been complied with all the relevant national regulations and institutional policies for the care and use of animals, and has been approved by the Laboratory Animal Welfare and Ethics Committee of the Army Medical University.

### 2.2 Induction of NeP

Mice were subjected to CCI surgery as previously described [3]. Briefly, under isoflurane (2%–4%) anesthesia, the right common sciatic nerve branches (close to the trifurcation into the sural, peroneal, and tibial nerve) were exposed and ligated with 4–0 cotton sutures. Mice without nerve ligation as the sham-operated group. Then, these CCI mice were divided into three groups: 1, 2, and 3 weeks.

### 2.3 Experimental process

**Figure 1:**
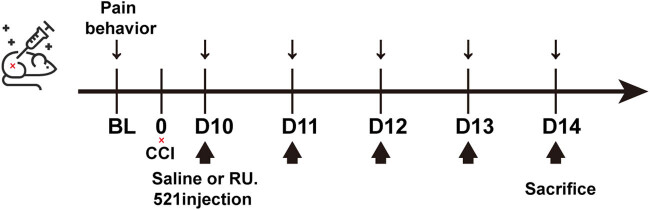
Timeline of RU.521 injection. The arrows indicate intraperitoneal injection of saline or RU.521 at a dose of 5 mg/kg/day, with thermal latency and mechanical thresholds measured before each injection, and euthanasia after testing on day 14.

The mice treated with the cGAS inhibitor RU.521 (Med Chem Express, USA) were randomly divided into four groups: (1) sham-operated; (2) CCI: CCI surgery group; (3) CCI + saline: CCI mice were intraperitoneally injected with 0.9% saline daily for 5 days; and (4) RU.521-treated CCI: CCI mice received intraperitoneal injection of RU.521 (5 mg/kg/day) for five consecutive days after 10 days of sciatic nerve ligation, and the determination of pain threshold was performed before administration each time. as depicted in Figure 1 (Figure 1). The dose and manner of injection of the drug were based on previous studies and our preliminary experiments [21]. Experimental mice were euthanized after completing treatment and tests on day 14.

### 2.4 Mechanical allodynia threshold and thermal latency measurement

The mechanical allodynia threshold of each group of mice was measured at 1 day before surgery and 3, 7, 14, and 21 days after surgery. Mice were placed independently in a cage for 30 min of acclimatization before measurement. Utilized von Frey filament to vertically stimulate the ipsilateral hind paw of mice, and recorded a positive response when the mouse withdraws its hind paw [22]. Measure again after 5 min, and the average value of the three measurements was taken as the paw-withdrawal mechanical threshold (PWMT).

The Hargreaves test was used to quantify the thermal threshold of radiant thermal stimulation in the hind paws of mice [22]. The radiation source was placed under the animal and aimed at the plantar, and the duration required for the mouse to withdraw its paw from the thermal stimulus was recorded as the paw withdrawal thermal latency (PWTL).

### 2.5 Cell culture and treatments

The mouse microglia (BV2) and neurons (Neuro2) were purchased from Procell Life Science & Technology Co., Ltd (Wuhan, China). Cells were cultured in DMEM medium (C11995500BT, Gibco, Thermo Fisher Scientific, USA) with 10% fetal bovine serum (9006-53-5, Biological Industries, Israel) and 1% penicillin/streptomycin (15140122, Gibco, Thermo Fisher Scientific, USA), incubated at 37°C with 5% CO_2_ and (Thermo Fisher Scientific, USA). Upon reaching a confluence exceeding 70%, BV2 cells were treated with mtDNA isolated from Neuro2 cells.

### 2.6 mtDNA isolation and transfection

According to the manufacturer’s instructions, Neuro2 cell mtDNA was isolated with the Mitochondrial DNA Isolation Kit (ab65321, Abcam, Britain). Briefly, cells were collected. The cells were resuspended in 1× cytoplasmic extraction buffer and incubated on ice for 15 min. The cells were homogenized and transferred to a centrifuge at 1,200×*g* for 10 min at 4°C. The supernatant was removed and resuspended pellets in 1× cytosolic extraction buffer and centrifuged again. The pellets (isolated mitochondria) were resuspended and lysed in mitochondrial lysis buffer on ice for 10 min, added to the enzyme mixture, and incubated for 60 min at 50°C. Subsequently, the samples were centrifuged and resuspended the particles (mtDNA) with Tris-EDTA (TE) buffer. The concentration of mtDNA was measured with a NanoDrop 2000 spectrophotometer (Thermo Fisher Scientific Rockford, USA).

According to the manufacturer’s instructions, Neuro2-derived mtDNA was transfected into BV2 cells using Attractene Transfection Reagent (301005, Qiagen, Germany). In brief, cells were seeded in six-well plates and reached a confluence of 70%. The mtDNA was diluted in TE buffer and mixed with serum-free cell culture medium to the desired concentration for the experiment and added the Attractene Transfection Reagent to the Eppendorf tube to promote the formation of transfection complexes. After vortexing, the samples were incubated at room temperature for 15 min. The transfection complexes were added to the plates and rotated to ensure uniform distribution.

### 2.7 Detection of mtDNA and cytosolic DNA with staining

The sciatic nerve and spinal cord of each group of mice were collected rapidly and preserved at −80°C. DNA was extracted according to the TIANamp Genomic DNA Kit (DP304, TIANGEN, Germany) manual. Quantitative PCR (qPCR) analysis was performed on the extracted DNA using primers specific for nuclear DNA (18S rDNA) and mitochondrial DNA (CO1 and ND1). The abundance of nuclear DNA, as detected by the 18S rDNA primers, was utilized as the internal control for standardizing mtDNA quantification. The primers used in this study are shown in Table 1.

**Table 1:** The primer sequences used for real-time PCR assay

Gene (mouse)	Forward primer sequence (5′–3′)	Reverse primer sequence (5′–3′)
IFN-beta	GAGGAAAGATTGACGTGGGAGAT	AGTCCGCCTCTGATGCTTAAAG
TNF-α	GCCTCCCTCTCATCAGTTCTATG	ACCTGGGAGTAGACAAGGTACAA
Actin, beta	ACTGTCGAGTCGCGTCC	CTGACCCATTCCCACCATCA
TLR9	ATGCCTTCGTGGTGTTCGAT	TCTCGGTCCTCCAGACACAA
cGAS	TCAGCTACCAAGATGCTGTCAA	AGTGTTACAGCAGGGCTTCC
NLRP3	TATCTCTCCCGCATCTCCATTTG	GCGTTCCTGTCCTTGATAGAGTA
18S rDNA	CCTGAGAAACGGCTACCACATC	CACCAGACTTGCCCTCCA
mt-CO1	GCATCTGTTCTGATTCTTTGGGCAC	GTGGTGGGCTCATACAATAAAGCC
mt-ND1	CGGCCCATTCGCGTTATTC	GATCGTAACGGAAGCGTGGA
Tert	CACGTACTGTATCCGCCAGT	TGCACTGGCATCTGAATCCT

Double-labeled immunofluorescence was used to show mtDNA released into the cytoplasm. The cells were incubated with 400 nM MitoTracker Red Stock Solution (M7512, MitoTracker™ Red CMXRos-Special Package, Invitrogen, USA) for 15–45 min under appropriate growth conditions in the dark. After the mitochondria were labeled with a MitoTracker dye, cells were washed in fresh pre-warmed growth medium and fixed with 4% paraformaldehyde for 15 min. Cell permeabilization and blocking were performed sequentially, as described in immunofluorescence staining. After incubation overnight at 4°C with the double-stranded DNA (dsDNA) primary antibody (1:100, sc-58749, Santa Cruz, CA, USA), the FITC-conjugated secondary antibody (ab150115, Abcam, Britain) was incubated for 1 h at room temperature. Finally, the cells were stained with DAPI and observed under Laser confocal microscopy (LSM780, ZEISS, Germany).

### 2.8 Quantitative polymerase chain reaction (qPCR) analysis

RNA was extracted from tissues or cells using RNA-Quick Purification kits (RN001, ES Science, Shanghai, China) and reverse transcribed into cDNA using Fast All-in-One RT Kit (RT001, ES Science, Shanghai, China) according to the instruction manual. The qPCR experiment was conducted in 10 μl of reaction mixtures containing 0.2 μl of forward primer, 0.2 μl of reverse primer, 1 μl of cDNA, 5 μl of the PowerUp™SYBR™ Green Master Mix (A25742, Thermo Fisher Scientific, USA), and 3.6 μl of RNase free ddH_2_O and quantified using a real-time PCR instrument (BIO-Rad, USA). As a part of the thermal cycling parameters, UDGase activation was performed for 2 min at 50°C and predenaturation for 2 min at 95°C, followed by 40 cycles for 1 s at 95°C and for 30 s at 60°C. To determine relative expression levels, the mRNA levels of each sample were normalized to those of Actin, beta mRNA by the 2^−ΔΔ^Ct method. Primer synthesis was performed by Beijing Tsingke Biotech Co., Ltd. (Beijing, China). The primers used in this study are shown in Table 1.

### 2.9 Western blotting analysis

Tissues and BV2 cells were collected and lysed in RIPA Lysis Buffer (P0013B, Beyotime, Shanghai, China) containing protease and phosphatase inhibitors for 15 min on ice; the mixture was centrifuged at 10,000×*g* for 15 min. The lysates were heated with sodium dodecyl sulfate-polyacrylamide gel electrophoresis Sample Loading Buffer 5× (P0015, Beyotime, Shanghai, China) for 10 min at 100°C. Then, the protein samples were electrophoresed on 10% polyacrylamide gradient SDS gels (PG212, Epizyme, Shanghai, China) and transferred onto polyvinylidene fluoride membranes (66543, Millipore, Billerica, MA, USA). The membranes were blocked in 5% bovine serum albumin (ST023-50g, Beyotime, Shanghai, China) and incubated overnight with primary antibodies against cGAS (31659, CST, MA, USA, 1:1,000), phosphorylated-STING (p-STING) (72971, CST, 1:1,000), STING (13647, CST, 1:1,000), ionized calcium-binding adaptor molecule 1 (IBA1) (382207, ZEN-BIOSCIENCE, Chengdu, China, 1:1,000), IRF3 (381333, ZEN-BIOSCIENCE, 1:1,000), GAPDH (60004-1-Ig, Proteintech, Wuhan, China, 1:5,000), and tubulin (66031-1-Ig, Proteintech, 1:5,000). Next, HRP-conjugated secondary antibodies were incubated for 1 h at room temperature. The immunoblots were observed in BeyoECL Moon (P0018FS, Beyotime, Shanghai, China) by BIO-Rad gel imaging instrument and quantified by ImageJ software (National Institutes of Health, USA).

### 2.10 Immunofluorescence staining

For fluorescence staining of tissue samples, freeze slices of mouse spinal dorsal cords at L3-L5 and sciatic nerve were used to permeabilize with 0.3% TritonX-100 and blocked with 10% goat-derived serum for 1 h at room temperature. Then, the slices were incubated overnight at 4°C with primary antibodies against dsDNA (sc-58749, Santa Cruz, USA, 1:100), cGAS (A23846, Abclonal, Wuhan, China, 1:100), GFAP (250027, ZEN-BIOSCIENCE, Chengdu, China, 1:100), Neuron (222545, ZEN-BIOSCIENCE, 1:100), and IBA1 (17198, CST, USA, 1:100). CoraLite488-conjugated Goat Anti-Mouse, CoraLite594-conjugated Goat Anti-Mouse, and CoraLite594-conjugated Goat Anti-Rabbit (1:100, Proteintech, Wuhan, China) antibodies were incubated for 1 h at room temperature. These slices were covered with DAPI (C1002, Beyotime, Shanghai, China) and photographed under Laser confocal microscopy (LSM780, ZEISS, Germany).

For fluorescence staining of cells, BV2 microglia were seeded in confocal dishes, fixed in 4% paraformaldehyde for 15 min, and permeabilized with 0.3% TritonX-100 for 20 min at room temperature. After blocking with 5% goat-derived serum, cells were incubated overnight with IRF3 (ZEN-BIOSCIENCE, Chengdu, China), IBA1 (1:100, Cell Signaling Technology, USA), and p-STING (PA5-105674, Thermo Fisher Scientific, USA, 1:100). Then incubated with CoraLite488-conjugated Goat Anti-Mouse, CoraLite594-conjugated Goat Anti-Rabbit, or Alexa Fluor^®^ 488 Goat Anti-Rabbit (1:100, Proteintech, Wuhan, China) antibodies for 1 h at room temperature, and DAPI staining solution was used for nuclear staining. Cells were observed under Laser confocal microscopy.

### 2.11 ROS determination

BV2 cells were seeded in 12-well plates and transfected mtDNA at different times. The DCFH-DA ROS probe was used to detect the production of intracellular ROS by the ROS Assay Kit (CA1410, Solarbio, Beijing, China), according to the manufacturer’s instructions.

### 2.12 Nitrate/nitrite measurement

BV2 cells seeded in six-well plates were transfected with mtDNA at 70% confluence. After stimulation ended, the cell culture supernatants were collected at 0, 3, 6, 12, and 24 h respectively, for the determination of Nitrate/Nitrite by the Total Nitric Oxide Assay Kit (S0023, Beyotime, Shanghai, China), according to the manufacturer’s instructions.

### 2.13 Statistical analysis

Statistical analysis was performed using GraphPad Prism 9.0 (San Diego, CA, USA). All data resulted from at least three independent experiments (*n* = 3) and presented as mean ± SEM. Before statistical analysis, All datasets were subjected to normality and variance homogeneity tests. When the data do not conform to the positive distribution or the variance is uneven, a nonparametric test is used. In addition, the same time point values of PWMT and PWTL among groups were analyzed by the application of one-way analysis of variance (ANOVA) with Tukey’s post hoc test. The remaining data were analyzed using either Student’s *t*-test or one-way ANOVA. The statistical significance was set to *P* < 0.05.

## 3 Results

### 3.1 CCI surgery downregulated mtDNA in the injured peripheral nerve and upregulated mtDNA in the spinal cord of CCI mice

**Figure 2:**
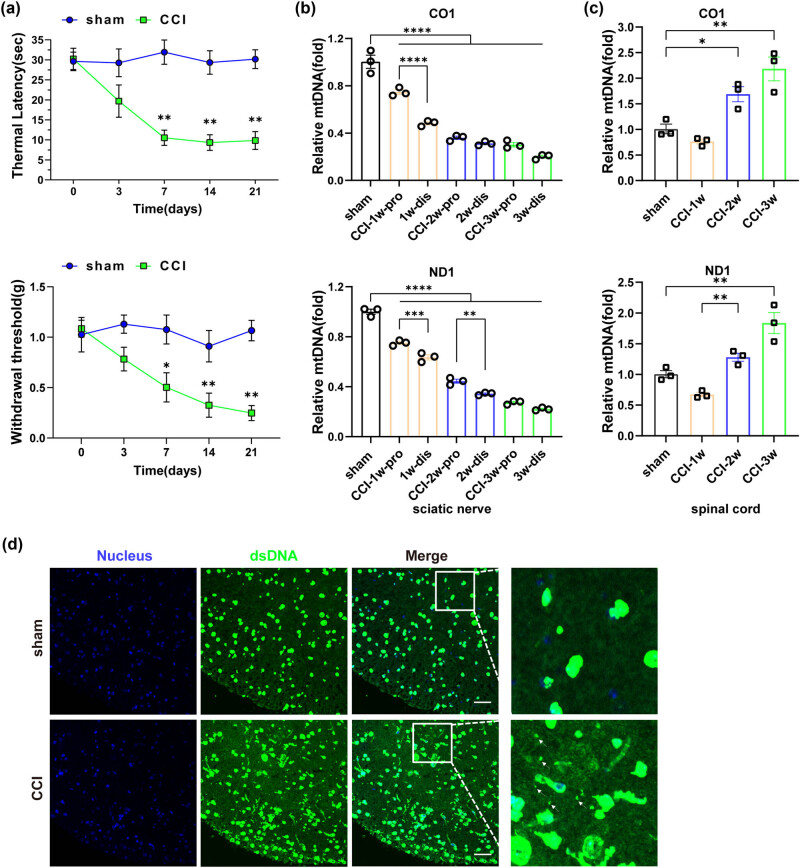
Changes in mtDNA content occurred in CCI mice following peripheral nerve injury. (a) Changes in mechanical threshold and thermal latency in mice (*n* = 3, *P < 0.05, **P < 0.01 compared to the sham group, the same time point values of PWMT and PWTL among groups were analyzed by the application of one-way ANOVA with Tukey’s post hoc test). (b) Changes in mtDNA content in the sciatic nerve by qPCR (*n* = 3, **P < 0.01, ***P <0.001, ****P < 0.0001, comparisons between the proximal and distal ends of the sciatic nerve within the same time group were performed using unpaired two-tailed *t*-test). (c) Changes in mtDNA content in the spinal cord by qPCR (*n* = 3, ***p = 0.001, ****P < 0.0001, using one-way ANOVA followed by Turkey’s test). (d) dsDNA in the spinal cord tissue of mice in each group was detected by immunofluorescent double-labeling with dsDNA labeled with anti-dsDNA (green), and nucleus labeled with DAPI (blue). The white arrow indicated free dsDNA, scale bar = 50 μM, and the framed image was amplified by four times.

As shown, on postoperative day 7, both PWMT and PWTL values of CCI mice were significantly lower compared to the sham-operated group (Figure 2a). DNA was extracted from the proximal and distal ends of the ligated nerve in mice, and qPCR was performed using CO1 and ND1 primers to assess mtDNA content. The results demonstrated a progressive decline in mtDNA content within the distal sciatic nerve of CCI mice, with a more pronounced decrease observed at each time point in the distal end compared to the proximal end (Figure 2b). Conversely, an increase in mtDNA content was observed in the spinal cord of CCI mice on the 14th day following CCI surgery (Figure 2c). Subsequently, we conducted co-immunostaining of dsDNA and the nucleus to further examine the changes in the spinal cord of CCI mice. In the sham-operated group, the spinal cord showed a well-defined texture with dsDNA staining largely overlapping with the nucleus. In contrast, the spinal cord tissue of CCI mice showed morphological blurring and disorganization, accompanied by numerous small extracellular dsDNA particles (Figure 2d), consistent with the qPCR results. Collectively, the data revealed a decrease in mtDNA content in the sciatic nerve and an increase in the spinal cord of CCI mice.

### 3.2 Activation of cGAS-IFN signaling in the spinal cord of CCI mice

**Figure 3:**
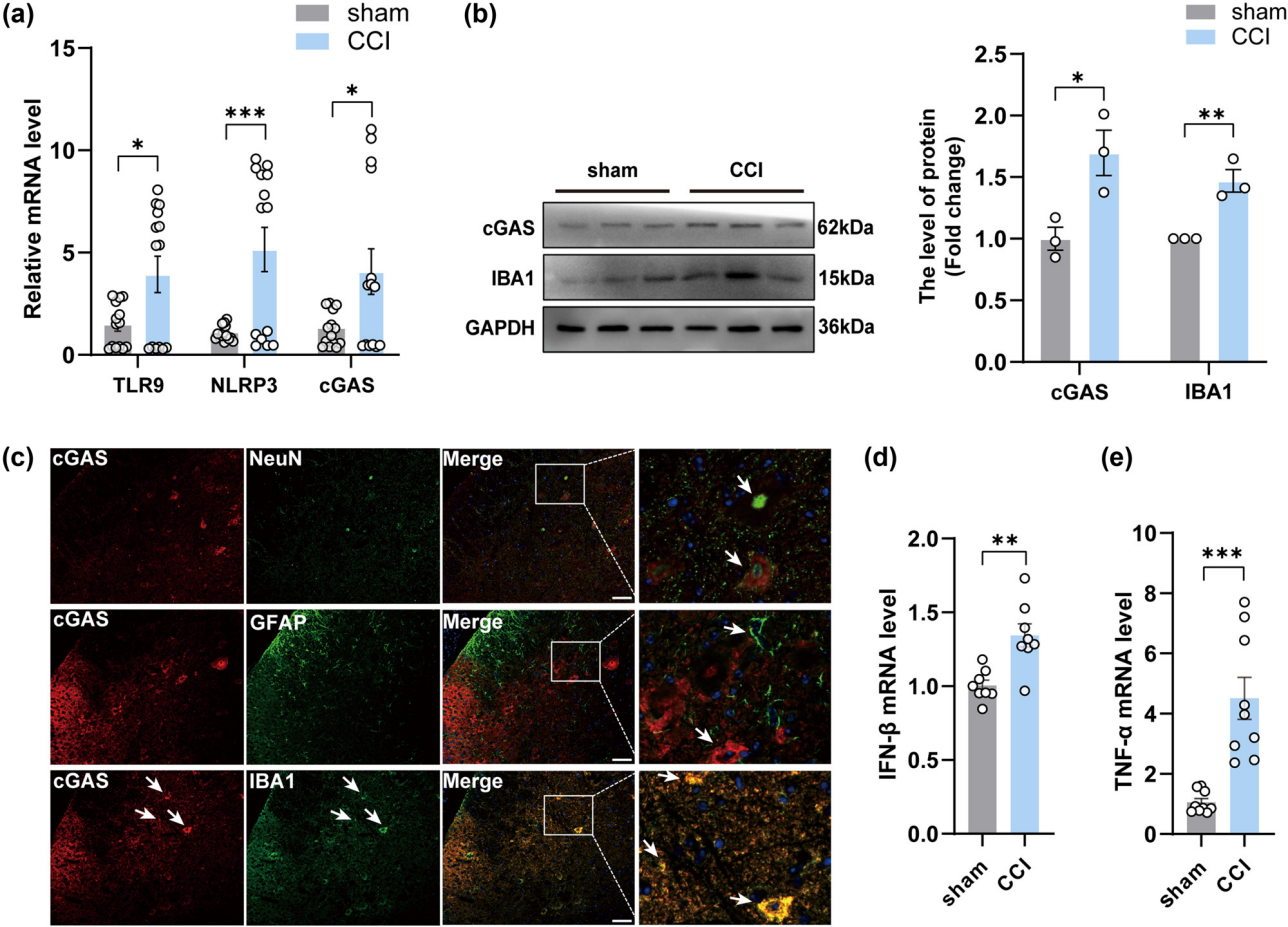
The activation of cGAS-IFN signaling occurred in the spinal cord of CCI mice. (a, d, and e) The mRNA expression levels of TLR9, NLRP3,cGAS, IFN-β and TNF-α in the spinal cord of groups (*n* = 3, **P < 0.01, ***P < 0.001, using unpaired two-tailed *t*-test). (b) The protein levels and quantification of cGAS in the spinal cord of mice in each group (*n* = 3, *P < 0.05, **P < 0.01 compared to the sham group, using unpaired two-tailed *t*-test). (c) Double immunofluorescence labeling of cGAS with GFAP (an astrocyte marker), NeuN (a neuronal marker), or IBA1 (a microglia marker) in the spinal cord of mice after CCI. scale bar = 50 μM, the framed image was amplified by four times.

Considering the significant increase in mtDNA in the spinal cord of CCI mice observed 14 days post-CCI surgery, we next began our investigation by quantifying the mRNA expression levels of receptor molecules associated with mtDNA at this time point, specifically TLR9, NLRP3, and cGAS. As shown, the expression levels of all these receptors were upregulated after surgery in the CCI group (Figure 3a). cGAS, a more specific intracellular DNA receptor, was examined in the spinal cord of CCI mice and showed significant upregulation in the second week after surgery (Figure 3b). We then performed immunofluorescence staining analysis to investigate the colocalization of cGAS within neurons, microglia, and astrocytes in the spinal cord. The results revealed a higher expression of cGAS in microglia compared to astrocytes and neurons (Figure 3c), aligning with the findings of Sun [21]. In addition, the expression of IBA1 in the spinal cord was increased (Figure 3b). Meanwhile, an increase in the mRNA expression of interferon-β (IFN-β) (Figure 3d), a molecule that plays a crucial role in facilitating endogenous immune responses and pro-inflammatory reactions. Similarly, upregulation of mRNA expression of the inflammatory mediator tumor necrosis factor-α (TNF-α) was observed in the spinal cord of CCI mice by qPCR analysis (Figure 3e). To sum up, these data suggested the upregulation of mtDNA-related receptors and the associated inflammatory response after CCI surgery, while highlighting the increased presence of cGAS protein in microglia.

### 3.3 cGAS inhibitors reversed decreased pain threshold and spinal cord inflammation in CCI mice

**Figure 4:**
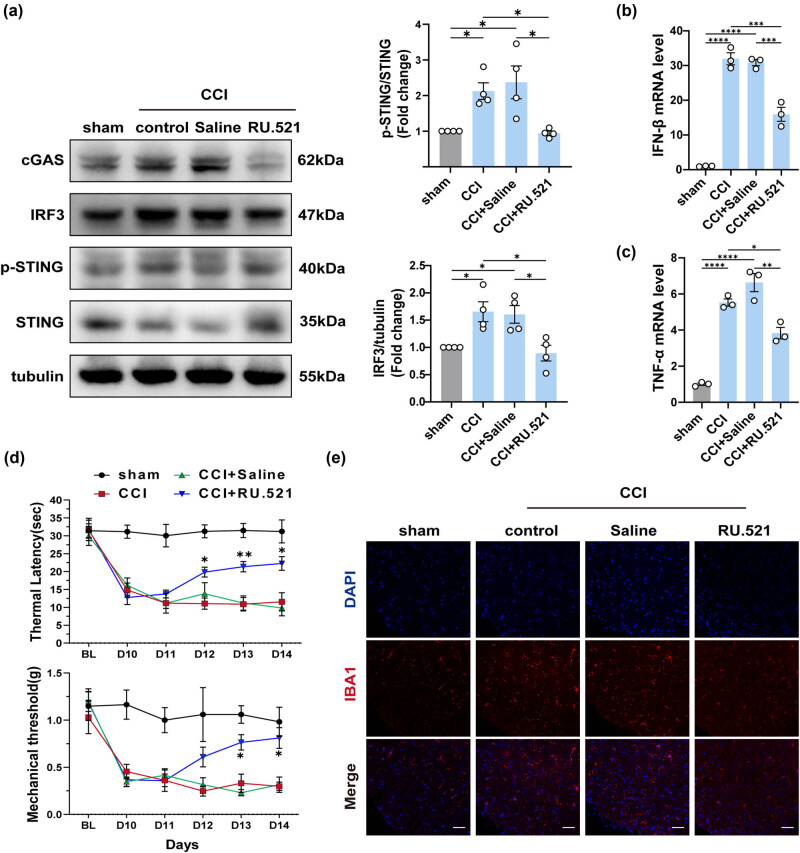
The pain threshold of mice with peripheral nerve damage is reversed by cGAS inhibitors. (a) The protein levels and quantification of cGAS, STING, IRF3, and p-STING in the spinal cord of each group (*n* = 4, *P < 0.05, **P < 0.01, using one-way ANOVA followed by Turkey’s test). (b and c) The mRNA levels of IFN-β and TNF-α in the spinal cord of mice in each group (*n* = 4, **P < 0.01, ***P < 0.001, ****P < 0.0001, using one-way ANOVA followed by Turkey’s test). (d) Thermal latency and mechanical thresholds were measured in each group of mice (*n* = 4, *P < 0.05, **P < 0.01 compared to the sham group, the same time point values of PWMT and PWTL among groups were analyzed by the application of one-way ANOVA with Tukey’s post hoc test). (e) Representative immunofluorescence images of IBA1 in the spinal cord of each group, *n* = 4, scale bar = 50 μM.

Studies have demonstrated that activation of the cGAS signaling induces the release of inflammatory factors and interferons. In addition, given the significant elevation of cGAS in the spinal cord of CCI mice observed in the above results, we investigated whether RU.521, a cGAS inhibitor, has therapeutic effects in NeP, and the mechanisms implicated. As depicted in the figure (Figure 4d), on the first day after injection (Day 11), there was no discernible improvement in thermal and mechanical allodynia in the CCI + RU.521 group. However, after three consecutive days of treatment, RU.521 partially reversed the decrease in PWMT and PWTL in the intervention group when compared to the untreated groups (CCI group and CCI + saline group). Pain thresholds in the saline-treated CCI mice group were not significantly different from those in the untreated CCI mice group. In addition, administration of RU.521 effectively suppressed the cGAS-IFN signaling pathway in CCI mice by inhibiting the expression of cGAS, phosphorylation of STING, IRF3, and IFN-β, which was confirmed by following the Western blotting and qPCR test (Figure 4a and b). Besides, inhibition of cGAS also led to a reduction in the expression of TNF-α (Figure 4c). Additionally, the alterations in fluorescence intensity observed in IBA1 staining indicated a significant reversal of microglial activation (Figure 4e). These data demonstrated that RU.521 effectively ameliorated allodynia and hyperalgesia in mice with NeP, indicating the involvement of the cGAS-IFN pathway in the pathogenesis of NeP.

### 3.4 Neuron-derived mtDNA activated BV2 microglial cells and promoted the release of inflammatory factors

**Figure 5:**
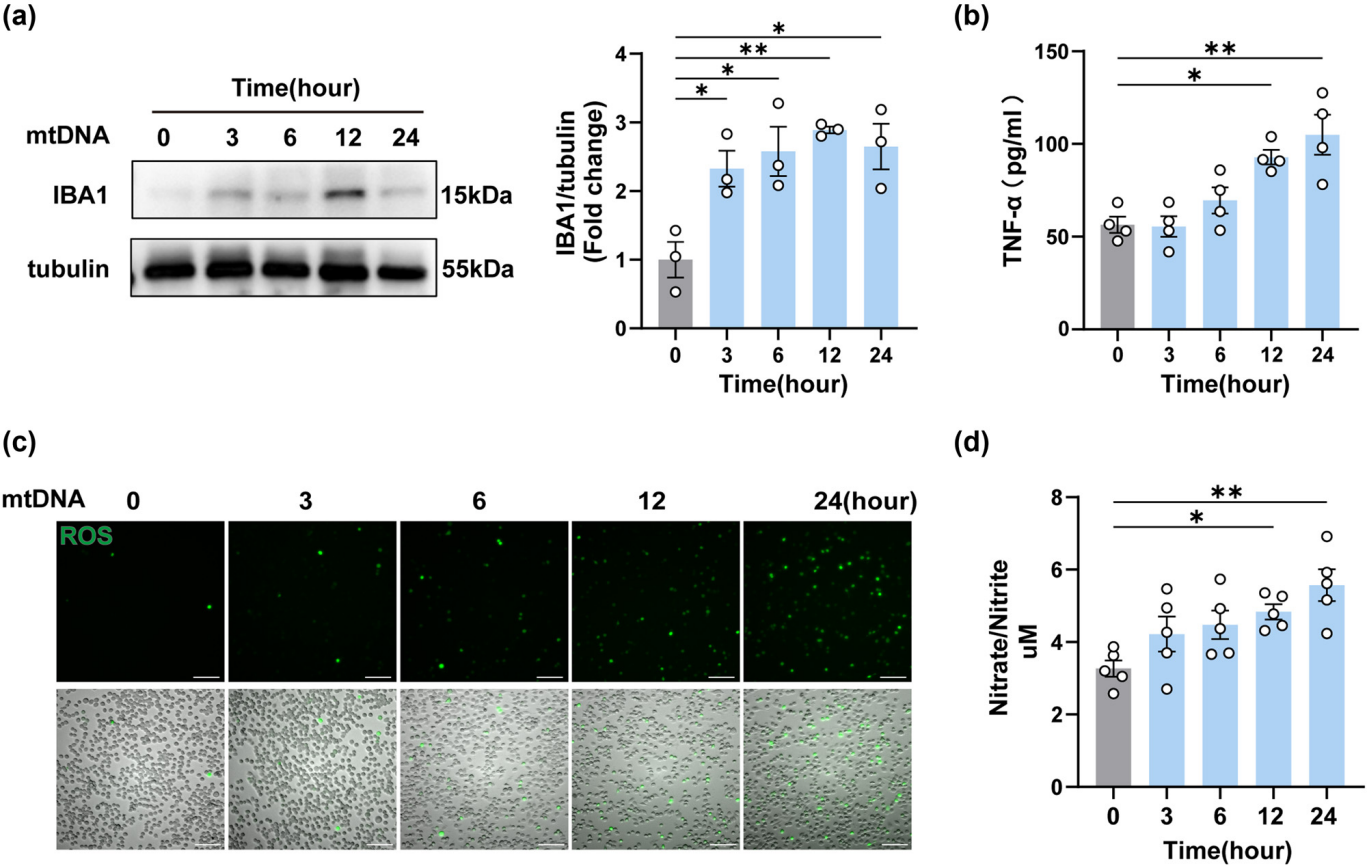
Neuron-derived mtDNA activated BV2 microglial cells and promoted the release of inflammatory factors. (a) The protein levels and quantification of IBA1 after mtDNA treatment on BV2 cells for different times (*n* = 3, *P < 0.05, **P < 0.01 compared to the 0-h group, using one-way ANOVA followed by Turkey’s test). (b) TNF-α in cell supernatants was detected by ELISA (*n* = 4, *P < 0.05, **P < 0.01, using one-way ANOVA followed by Turkey’s test). (c) Representative oxidative stress images of mtDNA-treated BV2 cells at different times, *n* = 3, scale bar, 100 μM. (d) The release levels of NO were detected in BV2 cells after mtDNA treatment (*n* = 5, *P < 0.05, **P < 0.01, using one-way ANOVA followed by Turkey’s test).

In view of the diversity of cells in the spinal cord of mice, and the uncontrollability of *in vivo* experiments, it was not possible to accurately assess the effect of mtDNA on microglia, so we embarked on an *in vitro* investigation using BV2 cells. After confirming that the stimulated cells release mtDNA into the cytoplasm or outside the cell (Figure S1). We isolated and purified mtDNA from neuro2 cells, transfected it into BV2 cells, and then detected the expression of IBA1 in BV2 cells following mtDNA transfection [23,24]. As shown, an initial upregulation of IBA1 was observed at the outset of transfection and confirmed by western blotting (Figure 5a). This was followed by a gradual increase in the concentration of TNF-α in the cell supernatant (Figure 5b). Meanwhile, ROS in the cell supernatant increased progressively over time (Figure 5c). In parallel, there was an increase in NO production, which is also a marker of microglial activation (Figure 5d). In summary, these findings suggested that mtDNA, when it enters BV2 cells, triggers the activation of these cells and the subsequent release of inflammatory factors.

### 3.5 Neuron-derived mtDNA activated the cGAS-IFN signal pathway in BV2 cells

**Figure 6:**
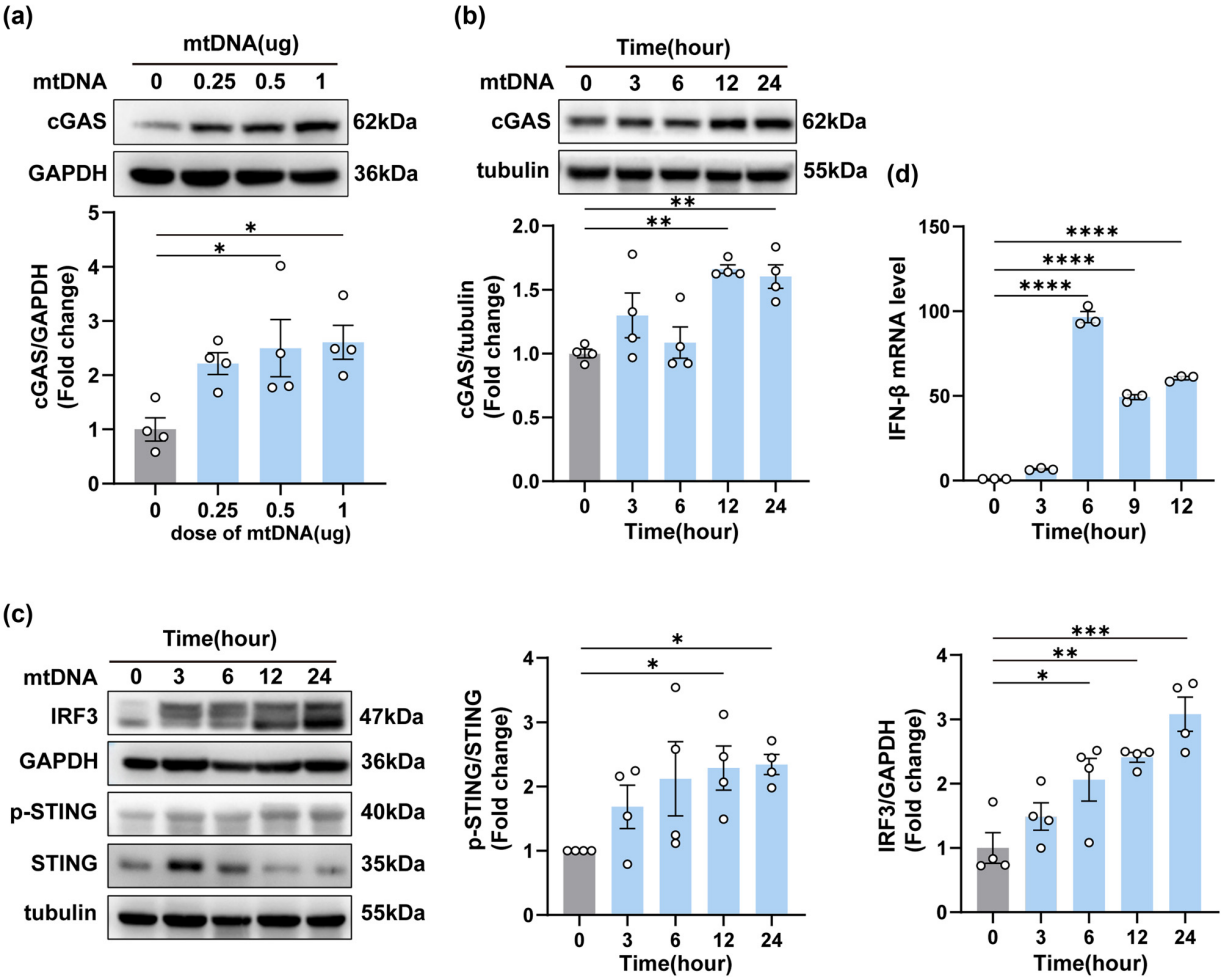
Neuron-derived mtDNA activated the cGAS-IFN signaling pathway in BV2 cells. (a) The protein levels and quantification of cGAS at different doses after the transfection of mtDNA (*n* = 3, *P < 0.05, using one-way ANOVA followed by Turkey’s test). (b and c) The protein levels and quantification of cGAS, IRF3, p-STING, and STING at different times after the transfection of mtDNA (*n* = 3, *P < 0.05, **P < 0.01, ***P < 0.001 compared to the 0-h group). (d) The mRNA expression levels of IFN-β (*P < 0.05, ****P < 0.0001, using one-way ANOVA followed by Turkey’s test).

To further reveal the underlying pro-inflammatory mechanism of neuron-derived mtDNA. We closely monitored the expression of cGAS protein after mtDNA transfection. Notably, there was a substantial activation of cGAS protein expression after transfection, and this activation exhibited a positive correlation with both the concentration of transfected mtDNA and the duration of transfection (Figure 6a and b). Moreover, we investigated the downstream elements of the cGAS signaling pathway at 0, 3, 6, 12, and 24 h in BV2 cells (Figure 6c). Notably, an interesting trend emerged: while the expression of STING showed a decrease, its phosphorylation state (p-STING) exhibited an increase. This was accompanied by elevated expression of IRF3 and IFN-β (Figure 6d). These results demonstrated that neuron-derived mtDNA activated the cGAS-IFN signaling pathway in BV2 cells.

### 3.6 cGAS inhibitors blocked mtDNA-induced inflammation in BV2 cells

**Figure 7:**
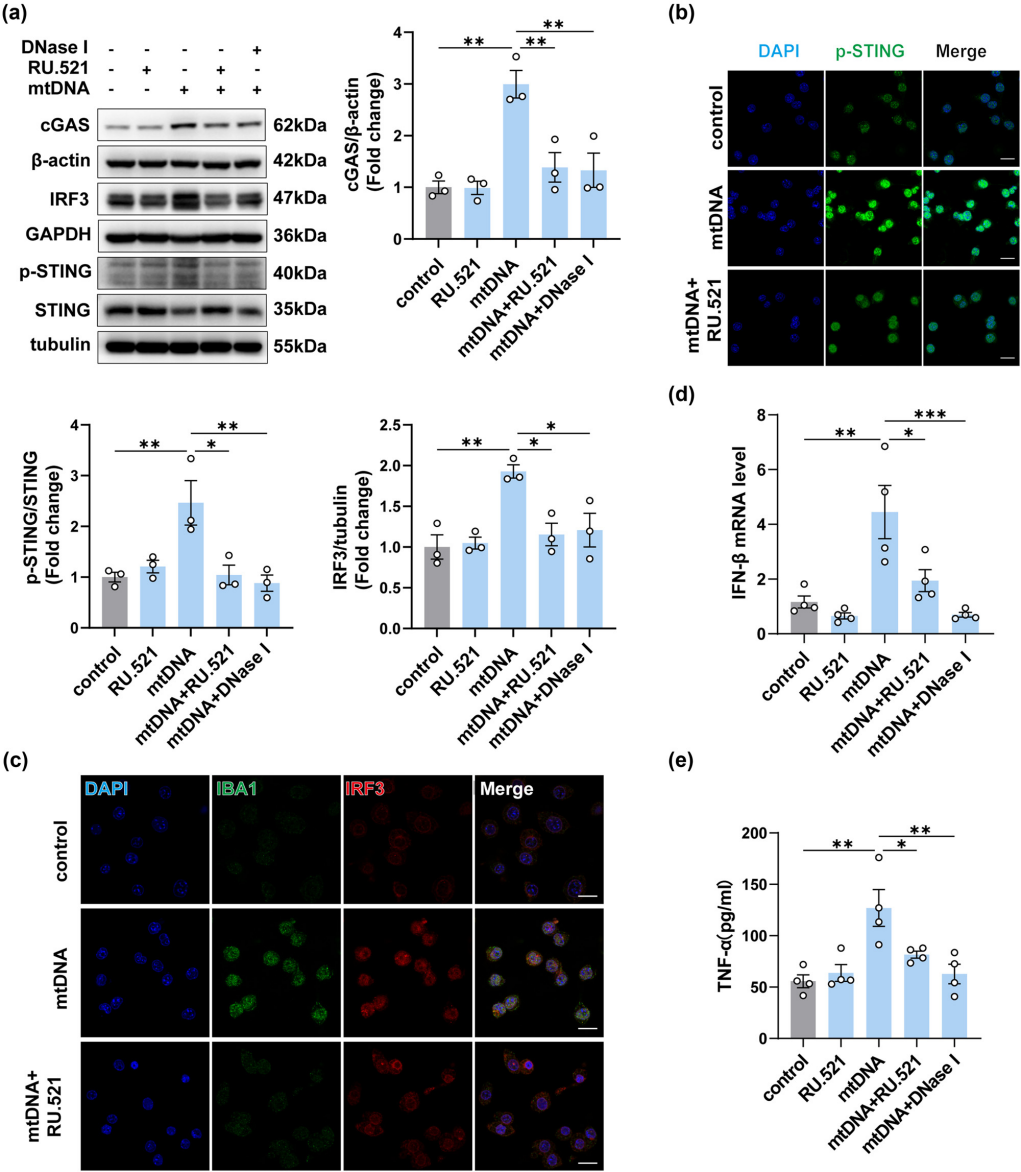
Inhibition of cGAS attenuated the inflammation in BV2 cells induced by neuron-derived mtDNA. (a) The protein levels and quantification of cGAS, STING, p-STING, and IRF3 in mtDNA-treated BV2 cells with or without intervention (*n* = 3, *P < 0.05, **P < 0.01, ***P < 0.001 compared to the control group). (b and c) Representative immunofluorescence images of IBA1, IRF3, and p-STING in each group, *n* = 3, scale bar = 20 μM. (d) The mRNA expression levels of IFN-β (*n* = 3, *P < 0.05, ***P < 0.001, using one-way ANOVA followed by Turkey’s test). (e) TNF-α in cell supernatants was detected by ELISA (*n* = 4, *P < 0.05, **P < 0.01, using one-way ANOVA followed by Turkey’s test).

To further confirm the role of cGAS in mtDNA-induced neuroinflammation, we used RU.521 to block the cGAS signaling pathway in BV2 cells. DNase I, an endonuclease, was employed to evaluate the efficacy of RU.521 by directly eliminating mtDNA [25,26]. Notably, there was a significant upsurge in cGAS expression in mtDNA-treated BV2 cells over 12 h. Pre-treatment with 10 μM RU.521 for 2 h effectively reversed the increase in cGAS expression (Figure 7a). We then detected the expression of phosphorylated STING and IRF3 in mtDNA-treated BV2 cells, comparing those pretreated or not with RU.521 or DNase I, by Western blotting and immunostaining (Figure 7a–c). Concurrently, we assessed the activated BV2 cells through immunofluorescence staining for IBA1 (Figure 7c). As shown, the degree of reduction in expression of phosphorylated STING, IRF3, and fluorescence intensity of IBA1 in mtDNA + RU.521 group was similar to the effect observed when mtDNA was hydrolyzed by DNase I before transfection. Notably, the mRNA expression of IFN-β (Figure 7d) and the release of TNF-α in cell supernatants in the mtDNA + RU.521 group also showed a significant reduction (Figure 7e). In light of these findings, it is reasonable to conclude that the inhibition of cGAS effectively attenuates mtDNA-induced inflammation in BV2 cells.

## 4 Discussion

In this study, we demonstrated the elevation of mtDNA, cGAS signaling, activated microglia, and inflammatory mediators in the spinal cord of CCI mice, while the opposite trend was observed in the sciatic nerve. Inhibition of cGAS effectively alleviated allodynia and hyperalgesia in CCI mice and also attenuated the cGAS-induced inflammatory response in the spinal cord. Furthermore, our results confirmed that mtDNA triggers microglial activation and upregulates cGAS and its downstream inflammatory mediators in BV2 cells. Importantly, these responses are reversible upon administration of DNase I or cGAS inhibitors.

The release of mtDNA from neurons and glial cells has been extensively implicated in various neurological disorders [8,27,28]. In this study, the detection results of mtDNA in the sciatic nerve of CCI mice showed a significant decrease compared to sham-operated mice, and the extent of the decrease showed an increasing trend over time (Figure 2b), reflecting the temporal course of the decrease in pain threshold. This highlights the important role of mitochondrial dysfunction in nerve damage as well. It has been reported that stimulation of mitochondria after neuronal injury can cause mitochondrial damage, resulting in the release or degradation of mitochondrial contents (including mtDNA), and even directly lead to neuronal death under overstimulation [29]. The comparison between the distal group and the proximal group at the same time point after nerve ligation confirmed that the distal group may experience a greater reduction in mtDNA due to more severe cellular energy metabolism damage. We also examined the changes in mtDNA content in the spinal cord of CCI mice. Interestingly, mtDNA content in the spinal cord of CCI mice began to increase on day 14 after surgery, and the increase was most significant on day 21. The trend of increased mtDNA in the spinal cord is consistent with the pain threshold curve in mice, suggesting a possible regulatory role of mtDNA in NeP.

In subsequent cell experiments, we further confirmed that neuro2 cells can release a substantial amount of mtDNA into the cytoplasm and cell supernatant upon stimulation (Figure S1). Thus, the source of increased mtDNA in the spinal cord of CCI mice does not exclude the possibility of the release from neurons within the spinal cord following peripheral nerve damage. Besides, MS is characterized by elevated mtDNA levels and a robust inflammatory response [30]. This is consistent with our observation of increased mtDNA in the spinal cord of CCI mice. Nevertheless, real-time tracking is essential to confirm the source of elevated mtDNA in the spinal dorsal. More research is needed in the future. Moreover, previous studies have indicated a reduction in mtDNA detected in the cerebrospinal fluid during the early stages of Alzheimer’s and Parkinson’s diseases [30]. This contrasts with our findings of increased mtDNA levels in the spinal cord of CCI mice, which may be attributed to differences in experimental models and detection timing. In general, understanding the distinct impacts of mtDNA in different diseases remains a critical challenge that requires further investigation.

Microglia, acting as the vanguard of immune defense during nerve inflammation and injury [31], also contribute significantly to neuroinflammation, particularly as prolonged nociceptive stimulation promotes the creation of an inflammatory microenvironment [32,33]. Moreover, the release of inflammatory mediators from the affected tissue, such as IFN, NF-κB, and TNF-α, culminates in the sustained onset of pain [34]. Thus, in our *in vitro* experiments, we employed microglia as the recipient cells to delve deeper into the potential pro-inflammatory effects of mtDNA-induced activation of cGAS signaling in these cells. Our data are consistent with prior research [10]. Additionally, the utilization of RU.521 inhibited the cGAS-IFN signaling pathway in CCI mice, diminished spinal neuroinflammation, and further relieved NeP in CCI mice. This suggests that mtDNA is involved in the development of neuroinflammation by activating microglia.

It is worth mentioning that heightened levels of mitochondrial-derived RNA fragments in the blood of stroke patients have been observed to modulate gene expression and immune response in CD14 monocytes, exerting a significant influence on both central and peripheral immune responses via cholinergic pathways [35]. Thus, it is plausible that mtDNA could initiate additional signaling cascades and potentially induce inflammation in the spinal cord or periphery through alternative cell types.

While TLR9 and NLRP3 show significant increases, the role of cGAS, a cellular DNA sensor, has been extensively documented in neurological injury and neurodegenerative diseases, yet remains understudied in NeP [36,37]. Therefore, we intend to further investigate whether cGAS has an effect on NeP and its mechanism of action. When cGAS interacts with mtDNA, it catalyzes the formation of cGAMP, a secondary messenger that transmits signals, activating downstream signaling molecules such as STING and IRF3 [38]. This, in turn, triggers the transcription of the type I interferon pathway and the induction of innate immunity [39]. This is consistent with the findings in our study, and the application of RU.521 effectively reduces downstream protein levels of p-STING and IRF3. Our findings suggest that mtDNA likely contributes to neuroinflammation by upregulating cGAS signaling in NeP models.

Finally, some investigators have found that small fragments of mitochondria-derived RNA can target cholinergic mRNA and control cholinergic responses like miRNAs, which are involved in neuroinflammation mediated by a variety of neurological diseases [40,41]. In addition, a large number of non-coding RNAs, including lncRNAs, circRNAs, and miRNAs, have been reported to alleviate neuroinflammation and nociceptive hypersensitivity caused by nerve injury by modulating gene expression in damaged nerves and are potential therapeutic targets for NeP [42]. Mitochondrial DNA also functions as a derivative of mitochondria. These findings imply the existence of numerous unexplored aspects of mtDNA involvement in the nervous system, which necessitate further exploration in future studies.

## 5 Conclusion

The results of our study reveal that peripheral nerve injury leads to the accumulation of mtDNA and activation of cGAS-IFN signaling in the spinal cord of CCI mice. This activation subsequently increases mRNA transcription associated with the type I interferon response, leading to the induction of inflammation in mice. These results clearly validate the occurrence of mtDNA-mediated cGAS-IFN signaling activation as a regulatory mechanism in the inflammatory processes associated with NeP. The confirmation of the above mechanism provides new therapeutic targets for the treatment of NeP.

## Supplementary Material

Supplementary Figure
