# Supplementary material for: Mitochondrial DNA drives neuroinflammation through the cGAS-IFN signaling pathway in the spinal cord of neuropathic pain mice
Source: Open Life Sci. 2024 May 31;19(1):20220872. doi: 10.1515/biol-2022-0872 (PMC11151397; doi:10.1515/biol-2022-0872)
Supplement: Supplementary Figure [file biol-2022-0872-sm.pdf]

Supplementary material

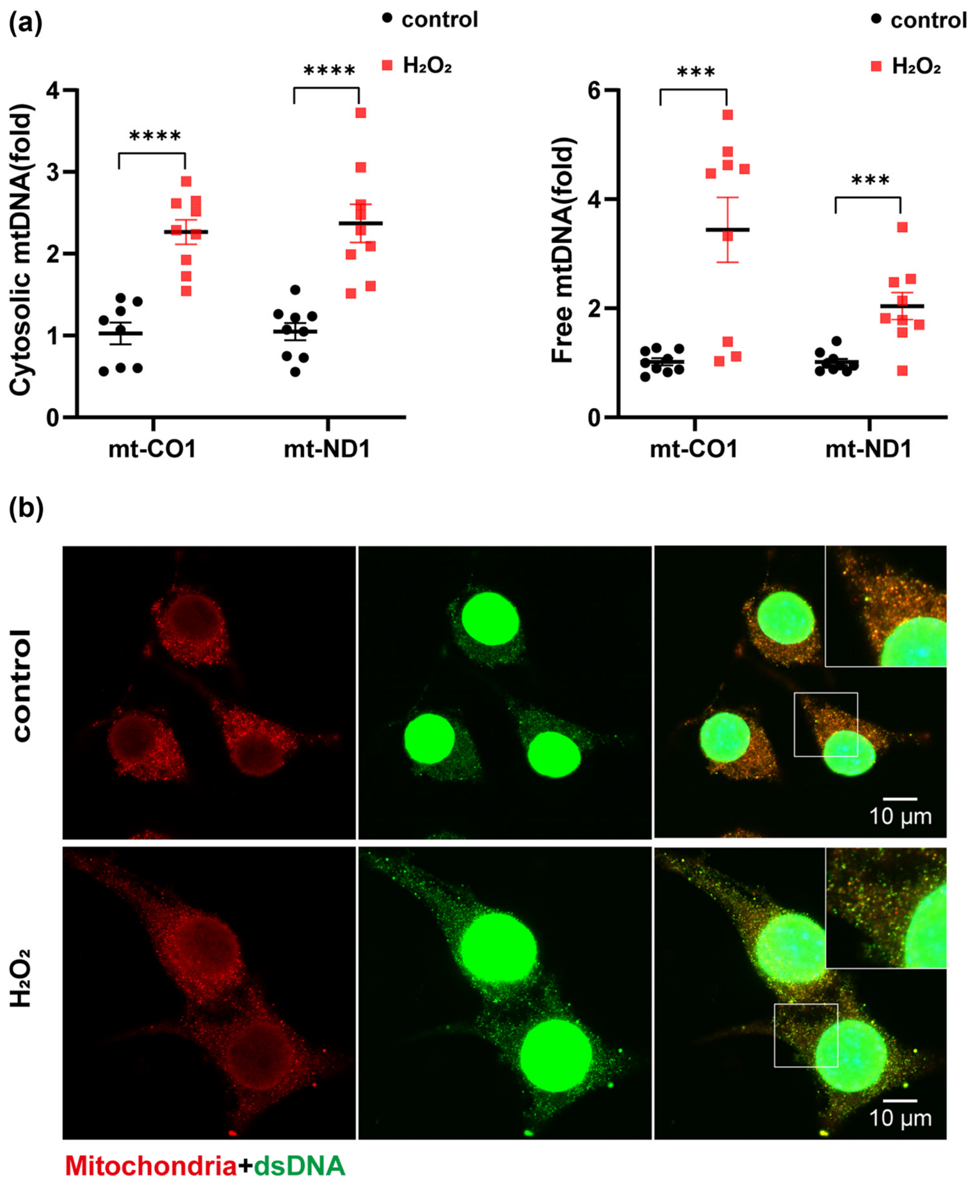

Figure S1: H_2O_2-induced the release of mtDNA in neuro2 cells. (a) mtDNA content in cytoplasm and cell supernatant was detected by qPCR (n = 3, ***P < 0.0005, ****P < 0.0001, using unpaired two-tailed t-test). (b) Mitochondria and dsDNA were analyzed by immunofluorescence co-localization in neuro2 cells, mitochondria were labeled with MitoTracker Red Stock Solution (red), dsDNA was labeled with anti-dsDNA (green), scale bar, 10 μM, (n = 3).
